# Evaluation of Changes in Metabolites of Saliva in Canine Obesity Using a Targeted Metabolomic Approach

**DOI:** 10.3390/ani11092501

**Published:** 2021-08-26

**Authors:** Alberto Muñoz-Prieto, Ivana Rubić, Anita Horvatić, Renata Barić Rafaj, José Joaquín Cerón, Asta Tvarijonaviciute, Vladimir Mrljak

**Affiliations:** 1 Clinic for Internal Diseases, Faculty of Veterinary Medicine, University of Zagreb, Heinzelova 55, 10000 Zagreb, Croatia; alberto.munoz@um.es (A.M.-P.); ivana.rubic94@hotmail.com (I.R.); vmrljak@vef.unizg.hr (V.M.); 2 Department of Chemistry and Biochemistry, Faculty of Food Technology and Biotechnology, University of Zagreb, Pierottijeva 6, 10000 Zagreb, Croatia; horvatic.ani@gmail.com; 3 Department of Chemistry and Biochemistry, Faculty of Veterinary Medicine, University of Zagreb, 10000 Zagreb, Croatia; rrafaj@vef.unizg.hr; 4 Interlab-UMU, Regional Campus of International Excellence “*Mare Nostrum*”, University of Murcia, 30100 Murcia, Spain; jjceron@um.es

**Keywords:** obesity, dogs, saliva, targeted metabolomics, biomarkers

## Abstract

**Simple Summary:**

The growing problem of obesity in dogs has become an epidemic that affects their quality of life. This study shows for the first time the changes in salivary metabolites in dogs with obesity using a targeted metabolomic approach. The analysis revealed alterations in 27 metabolites classified as amino acids, glycerides, sphingolipids, glycerophospholipids, and acylcarnitines. Some of the changes in the metabolites found in the saliva of obese dogs are associated with insulin resistance, which is usually related to obesity. These results indicate that saliva can show changes in its metabolites in canine obesity, making it a valuable source of biomarkers for this condition.

**Abstract:**

Obesity is a common problem in pet dogs, affecting half of the general population in some countries. Excess body weight causes several disorders and has a negative impact on dogs’ quality of life. The use of metabolomics allows the identification of metabolite traces from the metabolic pathways involved in pathological processes. This study aimed to evaluate salivary metabolite variations in dogs with obesity. The salivary samples of 19 dogs were analyzed using a targeted metabolomic approach, through which 234 metabolites were quantified. Of these, multivariate analysis identified 27 different metabolites altered in dogs with obesity compared with control dogs. These metabolites were mainly classified as amino acids, glycerides, sphingolipids, glycerophospholipids, and acylcarnitines. Some of the changes in these metabolites reflect the insulin resistance status related to obesity in dogs. Overall, it can be concluded that the salivary metabolome of obese dogs reflects the metabolic changes occurring in obesity and could be a source of potential biomarkers for this complex condition.

## 1. Introduction

Obesity has become a common problem in dogs, with a prevalence exceeding half of the general population in some countries [1]. It is defined as an excess of adipose tissue due to multi-causal factors, being the excess of food administration and a reduced physical activity the most important [2]. Obesity can be a predisposing factor for many metabolic and other diseases, and it has become a serious concern in veterinary medicine. Excess body weight may predispose dogs to diabetes mellitus (DM), cardiorespiratory disease, orthopedic disease, and urinary and reproductive disorders that negatively affect their quality of life and lifespan [3]. Most studies related to obesity have been focused on serum, where different biomarkers have been identified, such as adiponectin [4], cardiovascular indicators [5], inflammatory biomarkers [6], and biomarkers related to glucose homeostasis-metabolism [7,8].

However, other biofluids have been considered to be of interest in the study of obesity. For example, saliva has some advantages over serum, since it is easy to obtain and its sampling is stress-free and painless for dogs, allowing the easy collection of multiple samples [9]. In the case of canine obesity, saliva has been used to detect biomarkers such as adiponectin and insulin [10,11]. 

Metabolomics enables the simultaneous measurement of a high number of metabolites present in a biological sample and can reveal specific alterations in a pathophysiological process [12]. Untargeted metabolomics is defined as the comprehensive analysis of all the measurable analytes in a sample, including chemical unknowns [13]. Although it offers the opportunity for novel target discovery, the principal challenges of this approach lie in the protocols and time required to process the extensive amounts of raw data generated, the difficulty in identifying and characterizing unknown small molecules, and its bias towards the detection of highly abundant molecules [13]. By contrast, targeted metabolomics consists of identifying defined groups of chemically characterized and biochemically annotated metabolites. Thus, metabolites are determined based on internal standards through quantitative or semi-quantitative analysis. This approach can be used to link novel associations between metabolites with specific physiological states [14]. Metabolomics studies have been performed on obese dogs, focusing on their serum [15] and plasma [16]. However, to the best of the author’s knowledge, neither untargeted nor targeted metabolomics have been applied to the study of canine obesity-related metabolic changes in saliva. 

This study aimed to investigate the salivary metabolome of obese dogs through a targeted metabolomic approach.

## 2. Materials and Methods

### 2.1. Dogs

The dogs enrolled in this study were experimental Beagles from a colony of the University of Murcia (Spain). Nineteen castrated male Beagle dogs were selected and divided into two groups: obese and control. The obese dogs developed nutritional obesity before the beginning of this study as a result of being fed ad libitum over six months with a hyperenergetic commercial diet (metabolizable energy: 3740 kcal/kg) (Affinity Libra High Energy, Affinity Petcare, Barcelona, Spain) composed of 30% protein, 15% fat, 3% fiber, 8% ash, and 1.5% Omega 6. The ingredients were cereals (including 4% whole wheat and 4% corn), meat and animal by-products (6% chicken, 4% pork), byproducts of vegetable origin, oils and fats, vegetable protein extracts, and mineral substances. The nutritional additives were vitamin A, vitamin D3, vitamin E, iron sulphate monohydrate, potassium iodide, copper sulphate pentahydrate, manganous sulphate monohydrate, zinc sulphate monohydrate, sodium selenite, and antioxidants. Drinking water was available ad libitum. The physical parameters indicated that all dogs were overweight or obese, as defined by the 5-scale body condition score (BCS), according to which a score greater than four is globally recognized as an indicator of obesity [17]. The fat mass percentage was determined using a dilution of a single dose of deuterium oxide (275 mg/kg), as previously described [18]. Therefore, the inclusion criteria used for the dogs with obesity were: (1) BCS > 4, (2) presence of obesity for at least 12 months, and (3) absence of other evident organ-related or metabolic diseases. The absence of obesity-related metabolic dysfunction was supported by the biochemical data from plasma triglycerides, cholesterol, and glucose, following the criteria previously reported [19]. Other metabolic diseases such as hypothyroidism were ruled out, since the dogs had values for T4 and TSH that were not compatible with this disease. In addition, the biochemical analysis also included acute phase proteins (APP), such as C-reactive protein (CRP) and haptoglobin; total proteins, albumin, and globulins; liver enzymes, such as aspartate aminotransferase (AST), alanine aminotransferase (ALT), alkaline phosphatase (ALP), gamma-glutamyl transferase (GGT), and total bilirubin; and renal function parameters, such as urea, creatinine, and electrolytes.

### 2.2. Saliva Sampling Procedures

Saliva samples were obtained in all dogs by introducing a small sponge into the dog’s mouth until it was thoroughly moist. No dogs used in this study showed any clinical signs of periodontitis. Sponges were placed in collection devices (Salivette, Sarstedt, Aktiengesellschaft & Co, Nümbrecht, Germany) and stored with ice until their arrival at the laboratory, where they were centrifuged at 3000× *g* for 20 min at 4 °C. The saliva samples were transferred to a 1.5 mL Eppendorf tube and stored at −80 °C until the metabolomic analysis.

At the same time, a blood sample was taken from each dog to perform a biochemical analysis to rule out any organ-related disease. All dogs were subjected to a 12 h fast before the blood and saliva sample collections.

The procedures were approved by the University of Murcia’s ethics committees and the Ministry of Agriculture, Livestock, Fishing, and Aquaculture of the Region of Murcia (A13170503).

### 2.3. Mass Spectroscopy Analysis (FIA-MS/MS and LC-MS/MS)

Sample preparation was performed using the AbsoluteIDQ p400 HR kit (Biocrates Life Sciences AG, Innsbruck, Austria) according to the manufacturer’s instructions. In brief, 10 μL of each saliva sample, quality control samples, blank, and zero samples (PBS) was added to the plate containing the internal standard mix. The plate was then dried for 30 min with a vacuum manifold. The samples were derivatized using 50 µL of 5% derivatization solution (phenylisothiocyanate (PITC) in water: ethanol: pyridine (1:1:1, *v*/*v*/*v*)) for 20 min and subsequently dried for 60 min using a vacuum manifold. Metabolites were then extracted with 300 μL of 5 mM ammonium acetate solution in methanol by shaking for 30 min at 450 rpm, then eluted using a vacuum manifold. A total of 150 µL of extracts was transferred from the capture plate to another empty 96-deepwell plate and diluted with water (50:50 *v*/*v*) for the liquid chromatography–mass spectrometry (LC–MS) analysis. The original plate was diluted with 250 μL of FIA mobile phase (10 mL ampule Biocrates FIA mobile phase in 290 mL of methanol) for flow injection analysis–mass spectrometry (FIA–MS). Both the LC and FIA plates were securely covered with silicon mats and shaken for 5 min at 500 rpm before the analysis.

Metabolites were measured with a targeted metabolomics approach, using a Dionex Ultimate 3000 ultra-high-performance liquid chromatography (UHPLC) system coupled with a Thermo Scientific Q Exactive Orbitrap mass spectrometer with the LC-MS instrument methods and parameters provided along with the kit (Biocrates Life Sciences AG, Innsbruck, Austria). Metabolites were identified and quantified using a standard workflow, using the MetIDQ™ software provided by Biocrates Life Sciences AG, Innsbruck, Austria. 

### 2.4. Statistical Analysis

Differences between BCS and BF in obese and control groups were investigated using the Mann–Whitney test, and the results were expressed as the median and the inter-quartile range (IQR). A non-parametric distribution of the data was assumed due to the small sample size of the groups. All the analyses were conducted with GraphPad Prism software (Version 9, Windows system).

### 2.5. Bioinformatics

To ensure data quality, several statistical methods were applied to the AbsoluteIDQ p400 data (MetIDQ™ software results–exported data) to identify differences between the groups. Data processing, statistical analysis, and data visualization were performed using the MetaboAnalyst software (web version 5.0). The analysis included data normalization and transformation, followed by univariate statistics with significant testing. Of the 408 metabolites measured with the Biocrates p400 Kit, those that were not identified or whose concentration was below the limit of detection (LOD) were excluded from further analysis. Subsequently, missing value imputation was used to replace missing values by 1/5 of the minimum positive value for each variable while maintaining the overall data structure. The study data were further processed by a log2 transformation to correct heteroscedasticity and skewness and to improve interpretability and visualization. To identify metabolites that significantly differed between the groups, we performed a Student’s t-test. The resulting -log10 (*p*-value) values were displayed versus the means of metabolite fold changes (FCs) within individual measured metabolite concentrations in a Volcano plot. Only metabolites with *p*-values lower than 0.05 were reported. Diagnostic models were constructed using partial least squares–discriminant analysis (PLS–DA), and the quality of the model was evaluated using accuracy, R2, and Q2. The variable importance of projection (VIP) values was calculated from the PLS–DA model. Only metabolites with VIP values >1 were considered discriminants. By using hierarchical clustering analysis (HCA), we further observed whether these metabolites could define the dogs’ obese metabolic status based on their relative concentration. Subsequently, the set of statistically significant metabolites between the groups was submitted to metabolite set enrichment analysis (MESA), which was also performed using the MetaboAnalyst platform.

## 3. Results

### 3.1. Characteristics of Dogs

**Figure 1:**
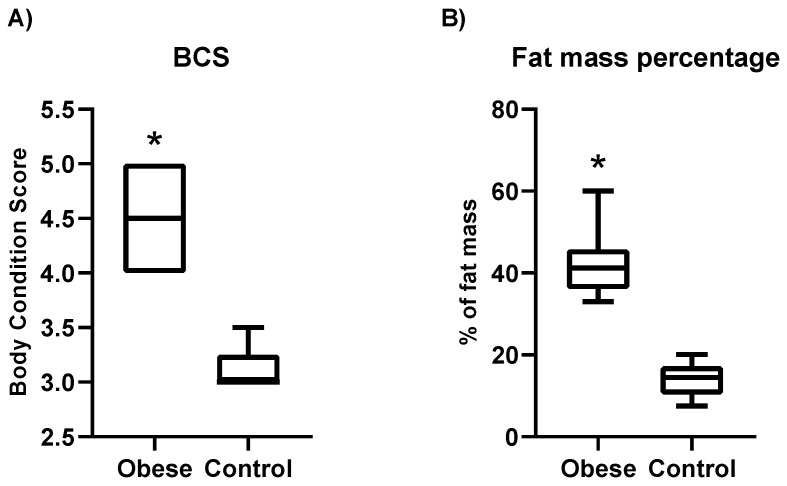
Changes in body condition score (BCS) (**A**) and body fat mass percentage among obese and control dogs (**B**). The plot shows the median (line within box), 25th and 75th percentiles (box), and the range (whiskers). Asterisks indicate significant differences between the obese group and the control. * *p* < 0.05; BCS: body condition score.

The group of obese dogs was integrated by ten dogs with a mean BCS of 4.5 (range: 4–5), with a mean body fat mass percentage of 42.13% (range: 33.04–47.60%), and a mean age of 5.2 years (range: 4–9 years). The control group was composed of nine dogs with a mean BCS of 3.1 (range: 3–3.5), with a mean body fat mass percentage of 13.80% (range: 7.5–20.10%), and a mean age of 5.8 years (range: 4–6 years). BCS and body fat mass were significantly higher in dogs with obesity than in control dogs (Figure 1). A complete set of the individual characteristics of the dogs is described in the Supplementary Material (Table S1).

### 3.2. Metabolomic Profile Differences between Obese and Control Dogs

**Figure 2:**
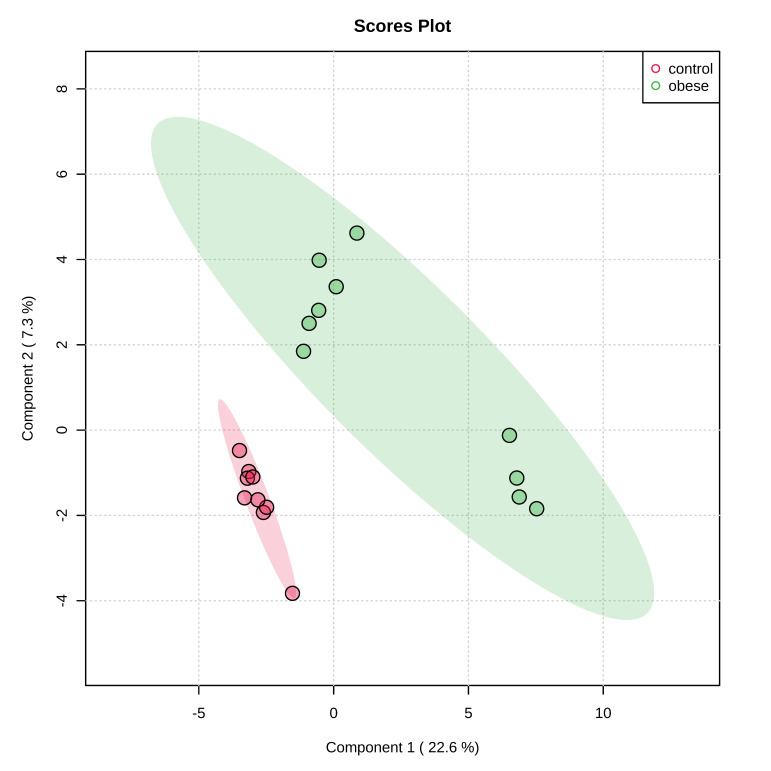
Partial least square-discriminant analysis (PLS–DA), showing a robust separation among the groups (control vs. obese). Each point in the plot corresponds to a saliva sample.

**Figure 3:**
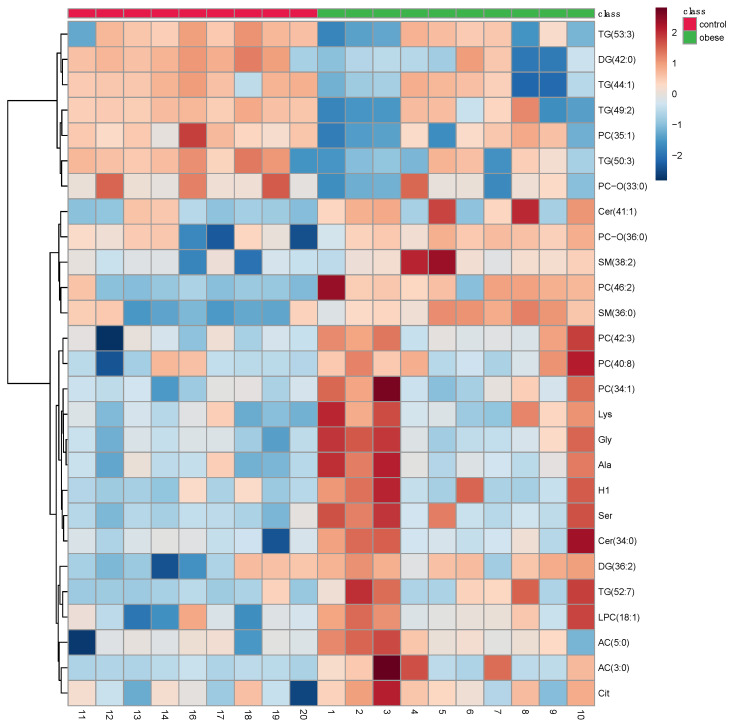
Hierarchical clustering differentiation of the 27 discriminative metabolites in obese and control dogs. Horizontal columns: relative concentration of each biologically relevant metabolite displaying distinct metabolic patterns between obese and control dogs. Each bar in the horizontal columns represents the expression intensity, where red bands indicate upregulated metabolites and blue bands indicate downregulated metabolites in the two groups.

The targeted metabolomics analysis allowed the identification and quantification of a total of 234 different compounds. Multivariate analysis using PLS–DA suggested a robust separation between the groups (accuracy = 0.63, R2 = 0.51; Q2 = 0.14) (Figure 2) that was also observed in the HCA (Figure 3).

**Figure 4:**
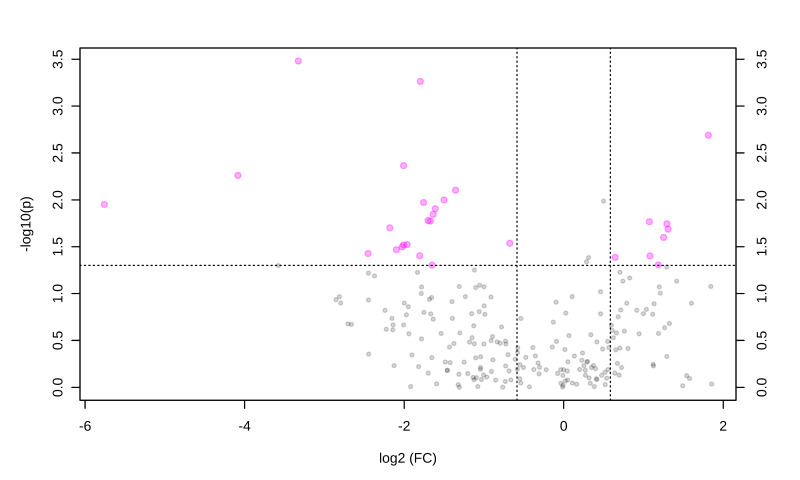
Volcano plot of the effect of obesity on salivary metabolites. Pink dots represent metabolites with significant differences and gray dots represent metabolites with no significant differences. The pink dots on the left represent metabolites above the thresholds whose concentrations were increased, while the pink dots on the right represent metabolites whose concentrations are decreased. The *x*-axis corresponds to log2 (fold change) and the *y*-axis to -log10 (*p*-value).

After multivariate statistics, the Student’s *t*-test and FC analysis were applied and depicted in a Volcano plot (Figure 4). This analysis highlighted 27 metabolites, including one sugar, five amino acids, seven glycerides, four sphingolipids, eight glycerophospholipids, and two acylcarnitines (ACs). A complete description of the significant metabolites identified is included in Table 1.

**Figure 5:**
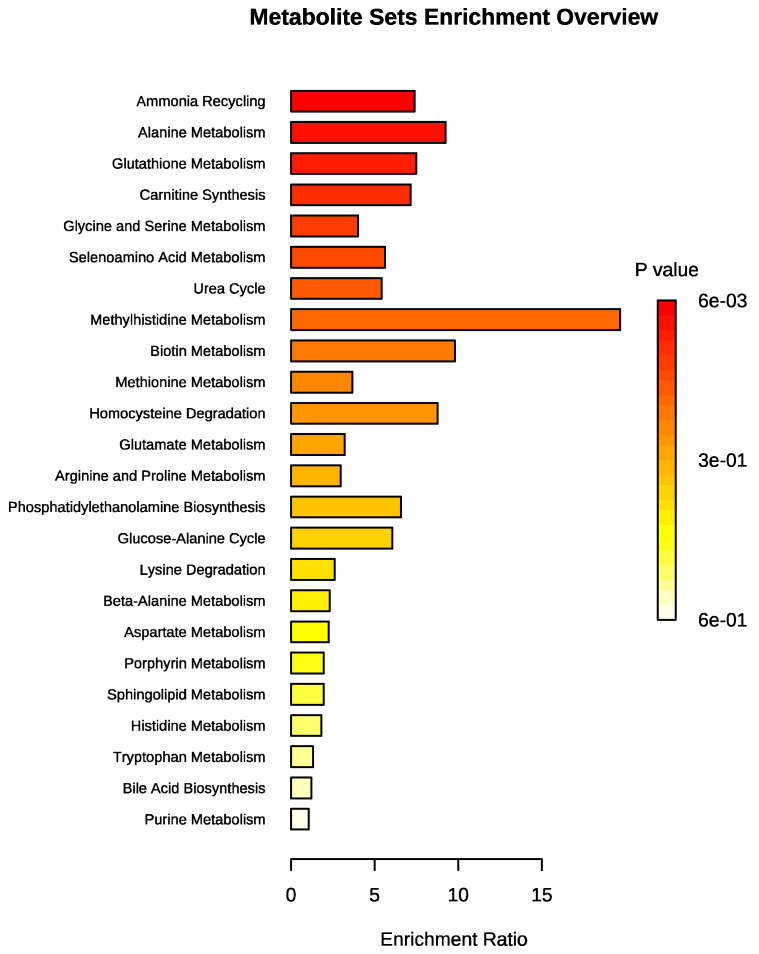
Metabolite set enrichment analysis (MSEA) of obesity. The horizontal bar graph summarizes 24 metabolic pathways using metabolites that were significantly altered in dogs with obesity.

The set of metabolites that were significantly different between the groups was subjected to pathway enrichment analysis to elucidate the metabolic pathways that were altered in obese dogs. This analysis showed a total of 24 pathways; the top 5 represented were ammonia recycling, alanine metabolism, glutathione metabolism, carnitine synthesis, and glycine and serine metabolism (Figure 5). A detailed analysis including all 24 identified pathways is provided in the Supplementary Materials (Table S2). 

## 4. Discussion

In this work, we describe for the first time the metabolic consequences of canine obesity in saliva using a targeted metabolomic approach. The mass spectrometric analysis of saliva revealed a significant alteration in metabolites related to amino acids, glycerides, sphingolipids, glycerophospholipids, and AC.

In our study, five amino acids were significantly changed in the salivary profile of obese dogs compared to normal-weight dogs. Specifically, the amino acid alterations were an increase in citrulline, serine, lysine, alanine, and glycine in obese dogs. In humans, increases in citrulline have been described in type 2 diabetes mellitus (T2DM) [20], which may also be a complication of obesity status in dogs. In the case of serine, it was positively correlated with body fat in humans [21]. Its increment in obese dogs could be related to body fat gain during the development of obesity. In this study, the obese dogs were obese for at least one year, and the upregulation in their salivary amino acids was still observed. This is in line with results previously reported in human serum samples, where increased amino acid concentrations were found in the serum of people with chronic obesity [21]. 

The different types of glycerides showed diverse behavior in the saliva of obese dogs. Some diglycerides (DG) and triglycerides (TG), such as DG (42:0), TG (44:1), TG (53:3), TG (50:3), and TG (53:6), decreased, but others increased, such as TG (52:7) and DG (36:2). Previous reports have indicated that obesity induces the elevation of selected components of glycerides and predisposes to T2DM [22,23]. However, we also found reduced levels of other types of glycerides in saliva. Therefore, additional studies are needed to elucidate the effects of obesity on lipid mechanisms affecting glyceride forms and their content in the saliva of obese dogs. 

Salivary sphingolipids were significantly increased in the obese group compared to the control. Sphingomyelin plays a role in cell apoptosis by its hydrolysis into ceramide, which is in turn responsible for the induction of apoptosis and involvement in inflammatory processes [24]. Ceramides are involved in insulin-mediated glucose uptake and are thought to play an essential role in the development of insulin resistance [25]. The increase in ceramides in the serum of people with obesity impairs glucose metabolism and predisposes one to T2DM [26]. In this report, we observed for the first time that sphingolipids such as ceramides are also altered in canine obesity, and that these changes are detected in saliva. Further studies would be of interest to determine the possible use of ceramide as a biomarker of insulin sensitivity disturbances. 

Phosphatidylcholines (PC) belong to the glycerophospholipids family, which are the main constituents of cellular membranes and are essential for cellular signal transduction [27]. In our report, various types of PC showed a different response in the saliva of obese dogs. Four PCs, (PC (46:2), PC (40:8), PC (42:3), and PC (34:1)), as well as a lysoPC called LPC (18:1), were found to be increased, while PC-0 (33:0), PC (35:1), and PC (38:2) were decreased. It has previously been shown that the biosynthesis of PCs was increased in a mouse model of obesity, and this increment was related to the inflammation of adipose tissue in obesity [28]. Furthermore, it has been shown that different phospholipids are increased in the serum of dogs with obesity [29]. Interestingly, decreased levels of PCs and their implication for the development of obesity-related T2DM in humans have also been reported [24]. Further studies are necessary to elucidate the cause of the different responses of PCs in the saliva of obese canines.

Finally, the two ACs found in our study were increased in the saliva of obese dogs compared to normal dogs. The ACs were positively correlated with the fat-free mass index in a previous study in humans [21]; furthermore, they were increased in obesity development and T2DM during pregnancy in women [30] and situations of obesity-related insulin resistance [24]. 

Although our targeted metabolomics approach revealed specific metabolites and pathways in canine saliva affected by obesity, this study has some limitations. We employed an experimental model of obesity in dogs. Although this allowed better control over experimental conditions, our findings should be confirmed through a clinical study of dogs with obesity. Furthermore, the diet used for the obesity development was a commercial formula and complete information about its ingredients and processing was not available. In addition, the dogs included in the study were all castrated males of only one breed. This precluded the possibility of studying the effect of sex hormones in the salivary metabolome of dogs. Further studies should also be performed with non-castrated animals of different breeds. Finally, this was a pilot study conducted using a small sample size, and the results should be interpreted with caution and validated with a larger number of samples. 

## 5. Conclusions

Obese dogs showed changes in their salivary metabolome under our experimental conditions. Namely, there was an increase in five amino acids (citrulline, serine, lysine, alanine, and glycine) that could be related to insulin resistance and obesity in dogs. Furthermore, alterations in lipids were also detected, with an upregulation of sphingolipids (e.g., ceramides) that may also be involved in the development of insulin resistance. Globally, it can be stated that the salivary metabolome of obese dogs can reflect the metabolic changes occurring during obesity status and could be a source of potential biomarkers for this condition.

## Figures and Tables

**Table 1:** Volcano plot data comparing obese versus control dogs. VIP values were obtained from a multivariate analysis using the PLS–DA model.

Metabolite Class	Metabolite	FC	Log2 (FC)	*p*-Value	-Log10 (*p*)	VIP Values	Regulation in the Obese Group
Sugar	H1	0.25	−1.96	0.02	1.52	1.17	Up
Amino acid	Citrulline	0.28	−1.80	0.03	1.40	1.41	Up
	Serine	0.24	−2.00	0.004	2.36	1.31	Up
	Lysine	0.32	−1.63	0.01	1.84	1.13	Up
	Alanine	0.31	−1.67	0.01	1.77	1.09	Up
	Glycine	0.32	−1.60	0.01	1.91	1.04	Up
Glycerides	TG(52:7)	0.05	−4.08	0.005	2.25	2.46	Up
	DG(42:0)	3.51	1.81	0.002	2.68	2.27	Down
	TG(44:1)	2.10	1.07	0.01	1.76	1.77	Down
	DG(36:2)	0.39	−1.35	0.007	2.10	1.72	Up
	TG(53:3)	2.11	1.08	0.03	1.40	1.53	Down
	TG(50:3)	2.47	1.30	0.02	1.68	1.32	Down
	TG(53:6)	2.27	1.18	0.04	1.30	1.16	Down
Sphingolipids	SM(36:0)	0.28	−1.79	0.0005	3.26	2.01	Up
	Cer(41:1)	0.22	−2.17	0.01	1.70	1.50	Up
	Cer(34:0)	0.24	−2.00	0.03	1.51	1.23	Up
	SM(38:2)	0.30	−1.70	0.01	1.77	1.15	Up
Glycerophospholipids	PC(46:2)	0.09	−3.32	0.0003	3.48	2.46	Up
	PC-O(33:0)	2.38	1.25	0.02	1.59	1.64	Down
	LPC(18:1)	0.29	−1.75	0.01	1.97	1.63	Up
	PC(35:1)	2.45	1.29	0.01	1.74	1.62	Down
	PC(40:8)	0.23	−2.09	0.03	1.46	1.57	Up
	PC(38:2)	1.56	0.64	0.04	1.38	1.41	Down
	PC(42:3)	0.35	−1.50	0.01	1.99	1.22	Up
	PC(34:1)	0.24	−2.02	0.03	1.49	1.11	Up
Acylcarnitines	AC(3:0)	0.01	−5.75	0.01	1.95	2.43	Up
	AC(5:0)	0.18	−2.45	0.03	1.42	1.8	Up

TG: triglycerides; DG: diglycerides; SM: sphingomielins; Cer: ceramides; PC: phosphatidylcholines; LPC: lysophosphadidylcholines; AC: acylcarnitines.

## Supplementary Materials

The following are available online at https://www.mdpi.com/article/10.3390/ani11092501/s1: Table S1: Individual characteristics of dogs included in the targeted metabolomic study. Table S2: Pathways enriched by significant metabolites in canine obesity.
