# Supplementary material for: Evaluation of Changes in Metabolites of Saliva in Canine Obesity Using a Targeted Metabolomic Approach
Source: Animals (Basel). 2021 Aug 26;11(9):2501. doi: 10.3390/ani11092501 (PMC8472812; doi:10.3390/ani11092501)
Supplement: Supplementary file 1 [file animals-11-02501-s001.zip › Supplementary Table S1.pdf]

Supplementary Table S1. Individual characteristics of dogs included in the targeted metabolomics study.

Group	Dog	Gender	Breed	Age (years)	BCS (1-5)	BF (%)
Obese	1	Male	Beagle	5	5	40.35
	2	Male	Beagle	6	5	33.04
	3	Male	Beagle	9	5	60
	4	Male	Beagle	5	5	42.15
	5	Male	Beagle	6	4.5	47.60
	6	Male	Beagle	4	4.5	39.70
	7	Male	Beagle	6	4	33.16
	8	Male	Beagle	7	4	43.03
	9	Male	Beagle	5	4	45.19
	10	Male	Beagle	4	4	37.11
Control	1	Male	Beagle	5	3	14.51
	2	Male	Beagle	4	3	17.13
	3	Male	Beagle	6	3.5	20.10
	4	Male	Beagle	5	3	12.45
	5	Male	Beagle	6	3	11.5
	6	Male	Beagle	6	3	7.5
	7	Male	Beagle	6	3	14.51
	8	Male	Beagle	5	3.5	17.3
	9	Male	Beagle	4	3	9.18
